# Provocation and localization of atrial ectopy in patients with atrial septal defects

**DOI:** 10.1007/s10840-022-01273-2

**Published:** 2022-06-23

**Authors:** Louisa O’Neill, Iain Sim, Daniel O’Hare, John Whitaker, Rahul K. Mukherjee, Steven Niederer, Matthew Wright, Vivienne Ezzat, Eric Rosenthal, Matthew I. Jones, Alessandra Frigiola, Mark D. O’Neill, Steven E. Williams

**Affiliations:** 1 Division of Imaging Sciences and Biomedical Engineering, King’s College London, 4thFloor North Wing, St. Thomas’ Hospital, London, SE1 7EH UK; 2 Bart’s Heart Centre, London, UK; 3 Guy’s and St Thomas’ NHS Foundation Trust, London, UK; 4 The University of Edinburgh, Edinburgh, UK

**Keywords:** Atrial septal defect, Right atrium, Atrial arrhythmia, Atrial ectopy

## Abstract

**Background:**

Atrial fibrillation (AF) is associated with atrial septal defects (ASDs), but the mechanism of arrhythmia in these patients is poorly understood. We hypothesised that right-sided atrial ectopy may predominate in this cohort. Here, we aimed to localise the origin of spontaneous and provoked atrial ectopy in ASD patients.

**Methods:**

Following invasive calibration of P-wave axes, 24-h Holter monitoring was used to determine the chamber of origin of spontaneous atrial ectopy. Simultaneous electrogram recording from multiple intra-cardiac catheters was used to determine the chamber of origin of isoprenaline-provoked ectopy. Comparison was made to a group of non-congenital heart disease AF patients.

**Results:**

Amongst ASD patients, a right-sided origin for spontaneous atrial ectopy was significantly more prevalent than a left-sided origin (24/30 patients with right-sided ectopy vs. 14/30 with left-sided ectopy, *P* = 0.015). Amongst AF patients, there was no difference in the prevalence of spontaneous right vs. left-sided ectopy. For isoprenaline-provoked ectopy, there was no significant difference in the proportions of patients with right-sided or left-sided ectopy in either group.

**Conclusions:**

When spontaneous atrial ectopy occurs in ASD patients, it is significantly more prevalent from a right-sided than left-sided origin. Isoprenaline infusion did not reveal the predilection for right-sided ectopy during electrophysiology study.

## Introduction

Atrial arrhythmias are common in patients with uncorrected secundum atrial septal defects (ASDs) [1]; however, arrhythmia mechanisms remain incompletely understood in this population. Although a common phenomenon in young asymptomatic subjects [2] and healthy elderly individuals [3], atrial ectopy can be associated with the initiation of atrial fibrillation (AF) [4]. In the absence of known AF, frequent atrial ectopy is predictive both of the development of AF and of adverse cardiac events including stroke, heart failure and death. [5]

In addition to pulmonary vein ectopy [6], non-pulmonary vein (PV) ectopy at sites including the superior vena cava, the crista terminalis and coronary sinus [7] is described and may be more common in the elderly, in females and in those with co-existent left ventricular impairment [8–10]. The site of origin of ectopy is of critical importance since the elimination of ectopy through ablation constitutes a cornerstone treatment for AF.

Little is known about sites of ectopy occurring in ASD patients. In a single epicardial mapping study of 10 patients, focal triggers and re-entrant circuits were confined to the right atrium in ASD patients with paroxysmal AF but were predominantly left atrial in those with persistent AF [11]. Nevertheless, marked right atrial structural remodelling occurs in ASD patients consisting of both atrial dilatation and atrial fibrosis [12, 13]. We hypothesised that right atrial ectopy may therefore predominate in ASD patients.

In this study, we sought to (1) characterise spontaneous atrial ectopy in ASD patients as right-sided or left-sided in origin, and (2) determine whether isoprenaline-infusion during electrophysiology study could be used to identify the dominant side of ectopy origin. Given the wealth of knowledge relating to non-PV ectopy in AF patients without ASDs, comparison is made to a control group consisting of non-congenital heart disease AF patients.

## Methods

The study conformed to the principles of Good Clinical Practice/The Declaration of Helsinki. Ethical approval was granted by the Health Research Authority (18/HRA/0083) for retrospective analysis of routinely collected clinical data and the London and Surrey Borders Research Ethics Committee (17/LO/1218) for prospective data collection. Written informed consent was obtained prior to participation.

### Non-invasive assessment of spontaneous ectopy

Adult patients with an uncorrected ASD, right atrial dilatation and previous 24-h Holter monitoring were included (ASD group). Comparison was made with non-congenital heart disease patients with paroxysmal AF and no history of ablation (AF group).

#### Holter P-wave axis calculation

**Fig. 1:**
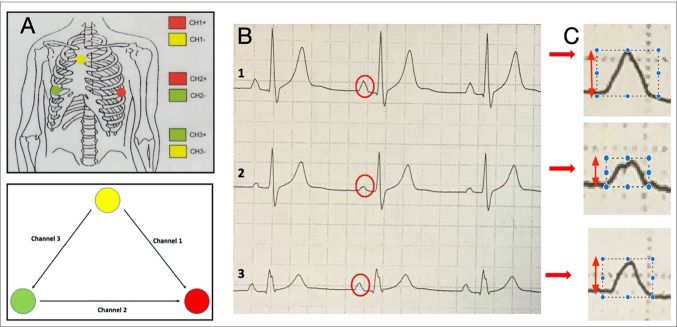
*Holter lead configuration and P wave angle calculation using a sinus rhythm example. ****A***;* Top panel shows Holter electrode placement with bottom panel depicting the three channels obtained, ****B***;* Example of Holter electrocardiograms obtained during sinus rhythm with P waves highlighted. ****C***;* Examples of how P wave amplitude was measured across all three P waves*

All atrial ectopic beats were identified on 24-h Holter monitoring using an automated algorithm. Representative atrial ectopic beats of all detected morphologies were then selected by an expert cardiac electrophysiologist, blinded to the existence of this study. Traces displaying the representative beats were digitised at 300dpi and used to measure the amplitude of positive and negative deflections of ectopic beats on each Holter channels Ch1–Ch3 (Fig. 1). Subtraction of the absolute value of the negative amplitude from the positive amplitude was performed to give a single value (${m}_{n}$) for each channel. Each channel was considered a vector together forming an equilateral triangle, with 0° defined as the position of Ch2 (lead I on the 12 lead ECG) and increasing angle values representing clockwise rotation. The $(x,y)$ components of the P-wave axis as recorded by each channel were calculated as: (${m}_{1}\mathrm{cos}60, {m}_{1}\mathrm{sin}60)$ for Ch1; $({m}_{2}\mathrm{cos}0, {m}_{2}\mathrm{sin}0)$ for Ch2; and $(-{m}_{3}\mathrm{cos}60, {-m}_{3}\mathrm{sin}60)$ for Ch3. P-wave vectors for every unique pairing of Holter leads were calculated ($Ch1+Ch2$; $Ch1+Ch3$ and $Ch2+Ch3$). The P-wave axis for each resulting vector was then calculated as $\theta ={\mathrm{tan}}^{-1}\left({~}^{\left|y\right|}\!\left/ \!{~}_{\left|x\right|}\right.\right)$.

Angles were adjusted to reflect the conventional notation of the cardiac frontal plane. The mean of the three angles obtained for each unique pair of Holter channels was calculated. When calculating the mean, if the P-wave axis determined by a single pair of Holter channels fell in an opposite-side quadrant to that from the other two Holter channel pairs, then that value was excluded from analysis. Similarly, if there was a > 45° difference between the P-wave axis for a single Holter channel pair and the next closest Holter channel pair, then that value was excluded.

#### Invasive determination of reference angles for P-wave origin

To provide reference angles using this approach to determine the origin of ectopic beats, an invasive validation protocol was performed using Holter recording during pacing in both ASD and AF patients. Right and left atrial intracardiac pacing was performed at multiple sites at a basic cycle length of 600 ms. Sites paced included the high and low lateral right atrial wall, the right atrial appendage and septum, the pulmonary vein ostia and the posterior left atrial wall. The axes of the Holter-recorded P-waves for each paced atrial site were calculated as described above and the results used to classify ambulatory ectopic beats as right- or left-atrial origin.

#### Site of origin of spontaneous ectopy

All atrial ectopic beats selected at the time of primary analysis were printed for each patient in both groups and analysed as described above.

### Invasive assessment of atrial ectopy

Adult patients > 18 years with uncorrected secundum ASD planned for closure were recruited. Comparison was made with a group of non-congenital heart disease patients with paroxysmal AF planned for first-time ablation.

#### ASD group procedural setup

Under ultrasound guidance, 8-Fr and 6-Fr vascular access sheaths were placed in the left femoral vein and an 8.5-Fr SR0 sheath (St. Jude Medical) was placed in the right femoral vein. Intravenous heparin (100 I.U./kg) was administered. A multielectrode mapping catheter (PentaRay, Biosense Webster) was introduced through the SR0 sheath to the left atrium through the ASD. An 8-Fr non-irrigated ablation catheter (Navistar, Biosense Webster) and a 6-Fr decapolar catheter (Inquiry, Abbott) were positioned in the right atrium and coronary sinus, respectively.

#### AF group procedural setup

Right femoral venous access was performed under ultrasound guidance consisting of two 8.5-Fr SR0 sheaths through which the PentaRay catheter and an irrigated ablation catheter (Thermocool Smarttouch, Biosense Webster) were introduced and one 6-Fr sheath through which the decapolar catheter was introduced into the coronary sinus. Single trans-septal puncture was performed, and heparin was administered to achieve an activated clotting time of 350 s.

#### Electrogram recording and analysis

**Fig. 2:**
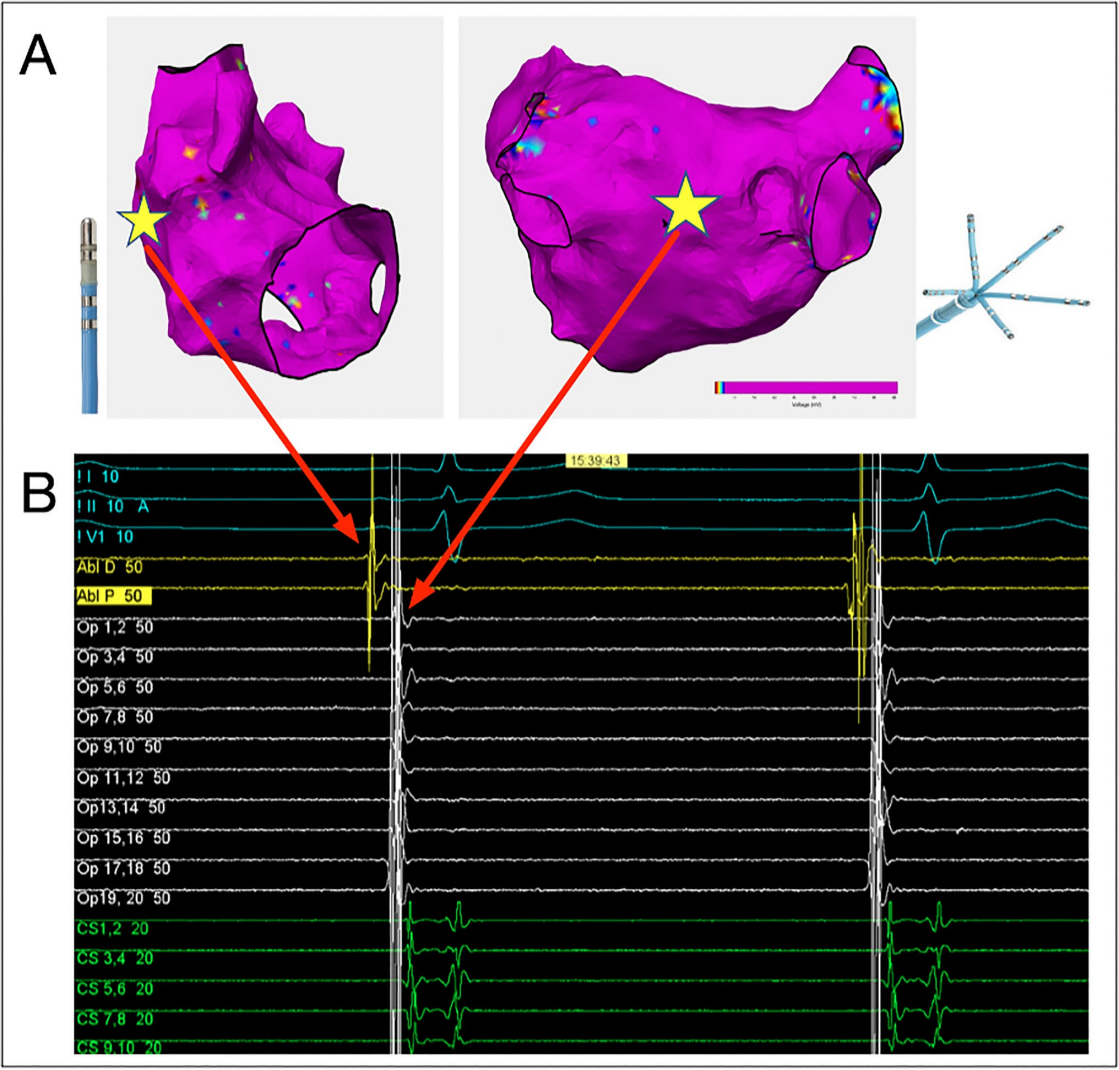
*Right and left atrial voltage map highlighting the position of the ablation catheter (left, yellow star) on the lateral right atrial wall and the Pentaray catheter on the posterior left atrial wall (right, yellow star) during isoprenaline infusion. ****B***; *Corresponding recording made during sinus rhythm at the start of the infusion. Blue electrograms represent surface ECG leads, yellow electrograms are recorded from the ablation catheter, white electrograms are recorded from the multipolar Pentaray catheter and green electrograms from the 318 coronary sinus decapolar catheter*

The PentaRay catheter was positioned against the posterior left atrial wall with splines splayed, the ablation catheter was positioned in cranial-caudal orientation against the lateral right atrial wall with good tissue contact on both bipolar electrode pairs (Fig. 2) and the decapole catheter was positioned in the coronary sinus with the proximal bipole pair at the coronary sinus. An incremental isoprenaline infusion (concentration 2 µg/ml, starting dose 5 µg/min) was commenced and increased by 5 µg/min every minute up to 20 µg/min. The maximum dose was continued for 2 min and the total infusion time was 5 min. If AF occurred during infusion, the initiating beat was analysed. Cardioversion was performed if AF did not terminate spontaneously in the ASD group. In AF patients, cardioversion was performed at the operator’s discretion. Intra-cardiac electrograms and surface ECG were recorded continuously during the infusion and for 2 min thereafter. The chamber of origin of all ectopic beats was determined based on the earliest activation across all intra-cardiac electrodes.

### Statistical analysis

Data analysis was performed using SPSS statistics (IBM, Version 24) and Prism (GraphPad Software, Version 7). Normality was assessed using the Shapiro–Wilk test with a *P*-value of < 0.05 indicating non-normally distributed data. Normally distributed continuous variables were expressed as mean ± standard deviation and non-normally distributed continuous variables as median and interquartile range. Comparison of means was performed using independent samples *T*-test for normally distributed data and Mann–Whitney *U* test for non-normally distributed data. The Fisher exact test was used to determine between group differences in categorical variables. *P* < 0.05 was considered statistically significant.

## Results

### Non-invasive assessment of atrial ectopy

#### Invasive determination of reference angles

**Fig. 3:**
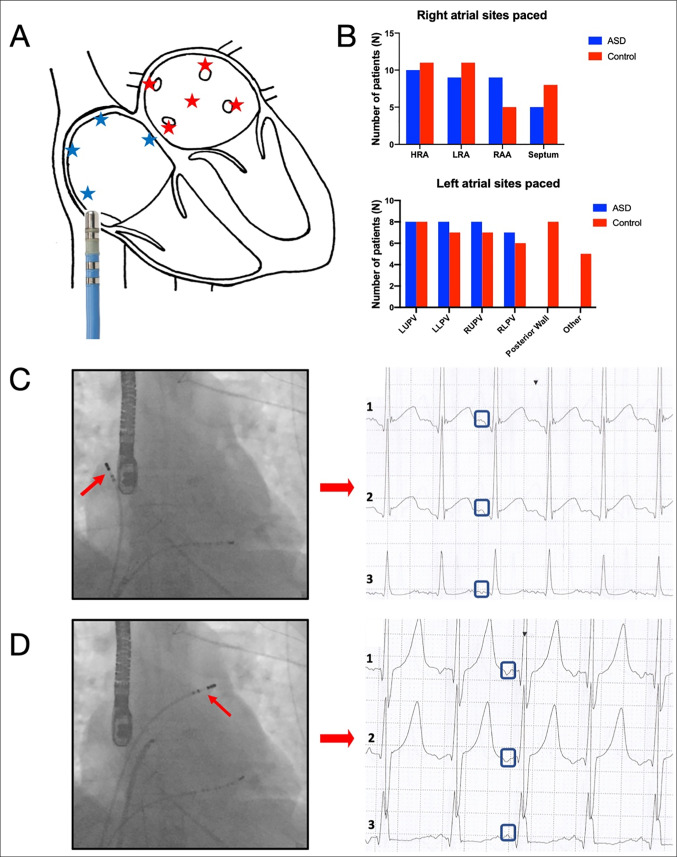
***A***;* Right and left atrial sites paced with the ablation catheter during the invasive validation protocol. ****B***; *Breakdown of right (top) and left (bottom) atrial sites paced in both study groups. ****C***; * Fluoroscopic image of ablation catheter at high lateral right atrial wall and corresponding Holter recording obtained during pacing at 600ms. *>***D***; * Fluoroscopic image of ablation catheter at ostium of left superior pulmonary vein and corresponding Holter recording obtained during pacing at 600ms*

**Fig. 4:**
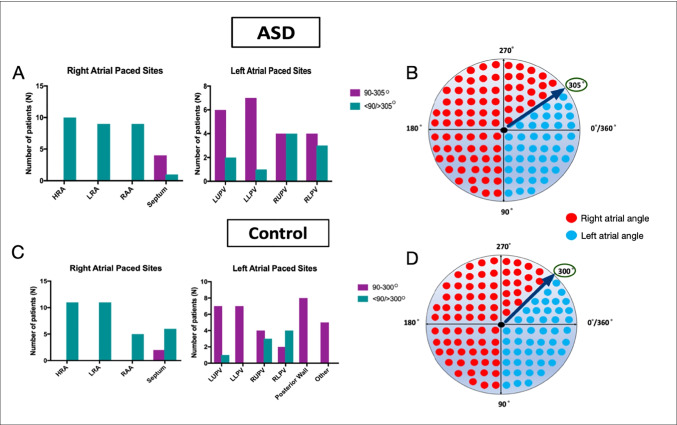
***A***; *Breakdown of angles falling between 90-300° vs <90/> 305° for each left and right atrial paced site in ASD patients. ****B***; *Graphical representation of angles described as either right or left atrial for the purpose of the retrospective study in ASD patients (based on analysis of atrial sites paced in the validation cohort).****C***;*Breakdown of angles falling between 90-300° vs <90/> 300° for each left and right atrial paced site in control patients. ****D***; *Graphical representation of angles described as either right or left atrial for the purpose of the retrospective study in control patients*

Ten ASD patients and 12 AF patients underwent multisite bi-atrial pacing during continuous Holter monitoring. Thirty-three right atrial and 33 left atrial paced Holter recordings were obtained in the ASD group and 35 right atrial and 41 left atrial paced Holter recordings were obtained in the AF group (Fig. 3). A P-wave vector between 90° and 305° had a 71% sensitivity and an 84% specificity for predicting a left atrial paced beat in the ASD group. In the AF group, a paced P-wave vector between 90° and 300° had an 81% sensitivity and a 94% specificity for predicting a left atrial pacing site. Accordingly, angles between 90° and 305° were considered left atrial and angles < 90° and > 305° were considered right atrial in the ASD group, whilst angles between 90° and 300° were considered left atrial and < 90° and > 300° were considered right atrial in the AF group (Fig. 4).

#### Retrospective Holter analysis

A separate cohort of 31 consecutive ASD patients and 35 consecutive AF patients were included in the analysis. Baseline characteristics are shown in Table 1. Patients in the ASD group were significantly younger than those in the AF group (*P* = 0.019).

**Table 1:** Baseline characteristics of ASD and AF patients undergoing evaluation of ambulatory atrial ectopy on Holter monitoring. *HTN* hypertension, *DM* diabetes mellitus, *TIA* transient ischaemic attack, *CCF* congestive cardiac failure, *CAD* coronary artery disease

	ASD (*n* = 31)	AF (*n* = 35)	*P* value
Age	53.6 ± 15.9	61.7 ± 10.6	0.019
Male sex (*n* (%))	15 (48.4)	21 (60)	0.344
RA area (cm^2^)	35.4 ± 9.7	23.9 ± 5	< 0.001
LA area (cm^2^)	28.7 ± 7.3	27.5 ± 5.3	0.593
HTN (*n* (%))	12 (38.7)	11 (31.4)	0.536
Diabetes (*n* (%))	2 (6.5)	1 (2.8)	0.484
Stroke/TIA (*n* (%))	1 (3.2)	1 (2.8)	0.931
CCF (*n* (%))	3 (9.7)	4 (11.4)	0.818
CAD (*n* (%))	2 (6.4)	5 (14.3)	0.302
CHADs VASc	1 (1–2)	2 (1–2)	0.904
Qp:Qs	2.4	n/a	
Known atrial arrhythmia (*n* (%))	7 (22.6)	35 (100)	< 0.001

Eighty-six representative, physiologist-selected premature atrial complexes were available for analysis in the ASD group, and 108 in the AF group with a median of 1 [1–4] and 2 [1–4] analysed premature atrial complexes per patient in the ASD and AF group, respectively (*P* = 0.439).

**Fig. 5:**
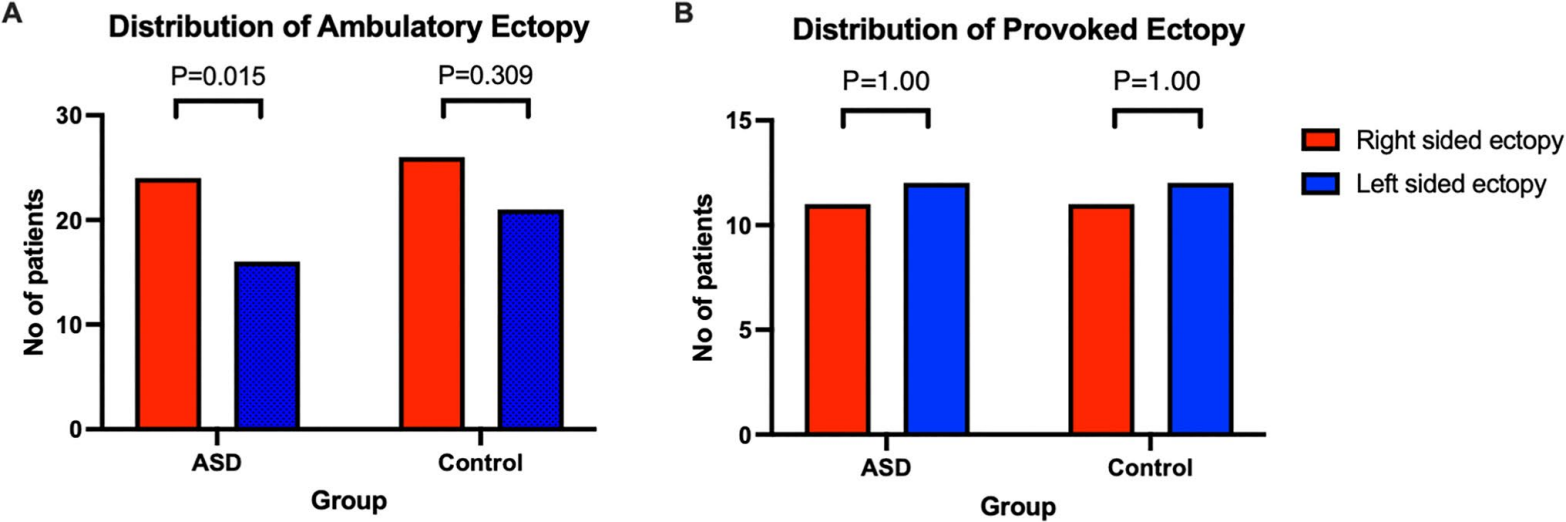
***A**** The percentage of patients with right vs left sided ectopy during 24-hour ambulatory Holter monitoring. ****B***; *The percentage of patients with provoked right vs left sided ectopy during isoprenaline infusion*

Amongst ASD patients, a right-sided origin for spontaneous atrial ectopy was significantly more prevalent than a left-sided origin (24/30 patients with right-sided ectopy vs. 14/30 patients with left-sided ectopy, *P* = 0.015, Fig. 5). Amongst AF patients, there was no difference in the prevalence of spontaneous right-sided vs. left-sided ectopy (26/35 patients with right-sided ectopy vs. 21/35 patients with left-sided ectopy, *P* = 0.309).

Over 24 h, the total number of premature atrial complexes was significantly lower in the ASD group than in the AF group (16 [4–69] vs 74 [21–465], *P* = 0.002). In the ASD group, premature atrial complex burden was associated with age (*R* = 0.411, *P* = 0.027) but no significant association was noted in the AF group.

### Invasive assessment of atrial ectopy

Seventeen ASD patients and 16 AF patients were included. Baseline characteristics are shown in Table 2. Heart rate increased by 79.1 ± 30.7% in the ASD group and 76.1 ± 29.7% in the AF group (*P* = 0.799) following isoprenaline infusion. Atrial ectopy was provoked in 16/17 ASD patients and 16/16 AF patients (*P* = 0.325). All induced ectopic beats were analysed. The total number of induced atrial ectopic beats and the number of ectopic foci were not significantly different between the ASD and AF groups (27 [4–57] vs 11.4 [5.5–63], *P* = 0.929 and 3 [1–3] vs 2 [1.25–3] *P* = 0.861, respectively).

**Table 2:** Baseline demographics of ASD and control patients undergoing incremental isoprenaline infusion. *RA* right atrial, *LA* left atrial, *HTN* hypertension, *DM* diabetes mellitus, *TIA* transient ischaemic attack, *CCF* congestive cardiac failure, *CAD* coronary artery disease

	ASD (*n* = 17)	AF (*n* = 16)	*P* value
Age	52.2 ± 10.4	57 ± 10.7	0.218
Male sex (*n*, %)	6 (35.3)	14 (87.5)	0.002
RA area (cm^2^)	36.1 ± 8.9	25.3 ± 6.2	0.006
LA area (cm^2^)	28.8 ± 4.6	26.9 ± 6	0.456
HTN (*n*, %)	3 (17.6)	4 (26.7)	0.606
Diabetes (*n*, %)	1 (5.9)	0 (0)	0.340
Stroke/TIA (*n*, %)	0 (0)	1 (6,7)	0.325
CCF (*n*, %)	0 (0)	0 (0)	
CAD (*n*, %)	0	2 (1.3)	0.133
CHADs VASc	1 (1–1)	1 (0–1)	0.533
Qp:Qs	2.3 ± 0.6	n/a	
Known atrial arrhythmia (*n* (%))	4 (23.5)	16 (100)	< 0.001
Number of ectopic foci induced per pt	3 (1–3)	2 (1.25–3)	P = 0.861

In the ASD group, right atrial ectopy was induced in 11 (65%) patients and left atrial ectopy in 12 (71%) patients (*P* = 1.0, Fig. 5). In the AF group, right atrial ectopy was also induced in 11 (69%) patients and left atrial ectopy in 12 (75%) patients (*P* = 1.0).

**Fig. 6:**
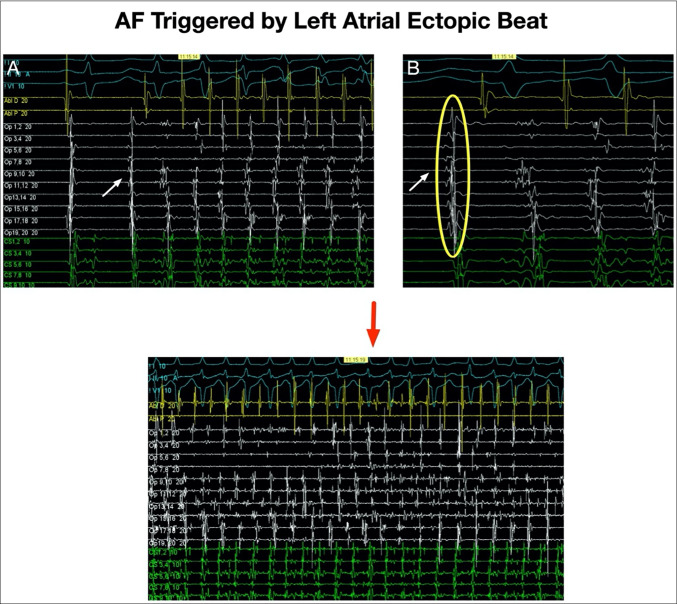
***A***; *Initiation of AF with a left atrial ectopic beat in a control patient. Earliest activation is seen on the Pentaray catheter indicated by the white arrow. ****B***;* close up view of same. ****C***; *AF continuing in the same patient*

AF was not induced in any ASD patient during the infusion; however, one patient experienced non-sustained atrial tachycardia triggered by right atrial ectopy at the end of the infusion. AF lasting > 30 s was induced in two patients in the AF group. In one patient, this occurred at the maximum infusion rate and was triggered by a left atrial beat (Fig. 6). In the second patient, AF occurred during the 2-min washout period and was also triggered by a left atrial beat. A third patient had a regular atrial tachycardia after completion of the infusion, also initiated by a left atrial ectopic beat.

## Discussion

The main findings of this study are (1) ambulatory atrial ectopy in ASD patients is significantly more prevalent from a right-sided than left-sided origin, (2) isoprenaline infusion did not reveal the predilection for right-sided atrial ectopy amongst ASD patients during electrophysiology study and (3) the chamber of origin of atrial ectopy may be classified from Holter monitoring with good sensitivity/specificity.

### Ambulatory ectopy

A greater prevalence of right- vs left-sided ambulatory ectopy was seen in the ASD patients studied here. This observation may reflect the predominance of right atrial remodelling which occurs in these patients. We have previously reported that right atrial fibrotic remodelling is identifiable by CMR imaging and is associated with the presence of atrial arrhythmia in ASD patients [13]. In non-congenital heart disease patients with AF, there is evidence to link fibrotic areas (identified by electroanatomic voltage mapping) and AF triggers [14]. Taken together, these findings suggest that PVI alone may not be sufficient for optimal AF management in the ASD cohort. This finding should be noted during the joint patient-physician decision-making process when considering the appropriateness of pulmonary vein isolation for the treatment of AF in ASD patients. Furthermore, we propose that adjunctive right ablation warrants future evaluation in larger scale trials, but we caution that the presented data alone do not yet support this intervention.

Prior studies have identified paroxysms of atrial ectopy as the initiating factor for over 90% of episodes of documented AF [4] and have demonstrated ectopic burden as a major risk factor for future development of AF [5]. In the patients studied here, there was an association between age and ectopy burden in the ASD group, supporting a previously described association between age and atrial arrhythmia prevalence in ASD patients [1, 15] This observation may be linked to progressive structural remodelling secondary to lifelong exposure to the ASD shunt.

This study presents a novel method for defining atrial ectopy origin from analysis of three Holter leads with validation against invasive pacing with good sensitivity and specificity for both patient groups. Prior studies evaluating P-wave morphology to determine origin of ectopy primarily relied on measurement of P-wave amplitude with visual assessment of P-wave morphology and polarity often on 12-lead ECG [16–18]. The approach employed here involves measurement of the amplitude and polarity of the ectopic beat providing quantitative analysis of the P-wave vector and predicted chamber of origin. Importantly, the P-wave vector ranges used to identify the chamber of origin were specific to the patient type as validation was carried out in both patient groups.

### Isoprenaline-induced ectopy

Isoprenaline is effective in inducing both adrenergic and vagally mediated atrial fibrillation [19] and may unmask atrial ectopic sites potentially guiding ablation [20]. Although several observational studies have reported favourable outcomes after ablation of inducible ectopy [21, 22], the utility of non-PV ectopy–based ablation is limited by inherent difficulties in the induction, mapping and eliminating of such ectopy [23, 24].

In this study, an equal distribution of right and left isoprenaline-induced ectopy was seen in ASD patients and AF patients, in contrast to the findings for spontaneous ectopy. Currently, there is no standardisation of isoprenaline provocation protocols nor is the relationship between isoprenaline dose and ectopy induction well described. Additionally, prior reports have shown a discrepancy in the prevalence of isoprenaline induced non-PV ectopy [25, 26] and it is uncertain if isoprenaline can reliably unmask all relevant ectopy [23]. In line with these observations, in this study, isoprenaline infusion did not reveal the predilection for the right-sided site of origin of ectopy identified in the non-invasive arm of the study.

Nevertheless, not all ectopic foci may be responsible for the initiation of AF with some sites more critical to the arrhythmogenesis than others [26]. In the AF patients studied here with sustained arrhythmia following isoprenaline infusion, the triggering beat was left atrial in all cases. Of interest, in the single ASD patient with inducible atrial arrhythmia, the triggering beat was right atrial in origin. However, as the number of patients with inducible arrhythmia were small, it is not possible to comment further on the utility of isoprenaline infusion for identifying the origin of atrial ectopy responsible for arrhythmogenesis in the ASD cohort studied. Interventional trials targeting isoprenaline-induced atrial ectopy are required in order to further understand its role in arrhythmogenesis in these patients and any potential benefit in eliminating this ectopy. The association between right atrial fibrotic remodelling and atrial arrhythmia in ASD patients should also be recognised [13] and it remains feasible that this remodelling is the critical factor for arrhythmogenesis in these patients rather than the site of origin of atrial ectopy.

## Limitations

The technique described in this study for classifying the site of origin of atrial ectopy from Holter recordings may be unlikely to distinguish between midline ectopic foci or between left and right atrial sites that lie in close proximity [18]. Our intention, however, was to broadly classify spontaneous ectopy as right- or left-sided, and according to the invasive validation, the sensitivity and specificity for this approach were good in both groups. The number of ASD patients with documented atrial arrhythmia was low limiting the ability to assess the association between the occurrence of ectopy and the initiation of arrhythmia. Additionally, in the absence of a follow-up period, the association between ectopy and the risk of development of AF cannot be determined. Future simulation and interventional studies will be required to fully evaluate the cause-effect relationship between ectopy and arrhythmia in patients with ASDs.

In the non-invasive arm, representative, physiologist-selected ectopic beats were studied, potentially providing a source of selection bias. However, the evaluated beats were selected in the same manner for all patients within each group and for both patient groups independently. Furthermore, selection of ectopic beats for disclosure was performed prior to the conception of this study and was therefore blinded to the inclusion of the data in this study. At the data analysis stage, all physiologist-selected ectopic beats were included. In the non-invasive arm, the ASD patients studied were significantly younger than the AF control group which may have influenced the findings.

## Conclusion

When spontaneous atrial ectopy occurs in ASD patients, it is significantly more prevalent from a right-sided than left-sided origin. Isoprenaline infusion did not reveal this predilection for right-sided ectopy during electrophysiology study. Further work is needed to fully assess the relevance of atrial ectopy origin to arrhythmogenesis in ASD patients.
